# A phase I study on the reversal of multidrug resistance (MDR) in vivo: nifedipine plus etoposide.

**DOI:** 10.1038/bjc.1992.53

**Published:** 1992-02

**Authors:** P. A. Philip, S. Joel, S. C. Monkman, E. Dolega-Ossowski, K. Tonkin, J. Carmichael, J. R. Idle, A. L. Harris

**Affiliations:** ICRF Clinical Oncology Unit, Churchill Hospital, Oxford, UK.

## Abstract

Multidrug resistance (MDR) is one of the mechanisms of resistance to multiple cytotoxic drugs and is mediated by the expression of a membrane pump called the P-glycoprotein. Nifedipine is one of the calcium channel blocking agents which reverses MDR in vitro. Fifteen patients with various malignancies received nifedipine at three dose levels: 40 mg, 60 mg and 80 mg orally twice daily for 6 days. Etoposide was administered intravenously on day 2 in a dose of 150-250 mg m-2 and orally 150-300 mg twice daily on days 3 and 4. Cardiovascular effects of nifedipine were dose limiting and the maximum tolerated dose was 60 mg bid. Mean area under the plasma concentration curve (AUC0-00) and plasma half-life (beta) of nifedipine and its major metabolite MI at the highest dose level were 7.87 microM.h, 7.97 h and 4.97 microM.h, 14.0 h respectively. Nifedipine did not interfere with the pharmacokinetics of etoposide.


Br. J. Cancer (1992), 65, 267–270 © Macmillan Press Ltd., 1992

### A phase I study on the reversal of multidrug resistance (MDR) *in vivo*: nifedipine plus etoposide

P.A. Philip[1], S. Joel[2], S.C. Monkman[3], E. Dolega-Ossowski[2], K. Tonkin[1], J. Carmichael[1], J.R. Idle[3] & A.L. Harris[1]

[1]*ICRF Clinical Oncology Unit, Churchill Hospital, Oxford OX3 7LJ;* [2]*Department of Medical Oncology, St Bartholomews and Homerton Hospitals, London E1; and* [3]*Department of Pharmacological Sciences, University of Newcastle upon Tyne, Newcastle upon Tyne NE2 4HH, UK.*

**Summary** Multidrug resistance (MDR) is one of the mechanisms of resistance to multiple cytotoxic drugs and is mediated by the expression of a membrane pump called the P-glycoprotein. Nifedipine is one of the calcium channel blocking agents which reverses MDR *in vitro*. Fifteen patients with various malignancies received nifedipine at three dose levels: 40 mg, 60 mg and 80 mg orally twice daily for 6 days. Etoposide was administered intravenously on day 2 in a dose of 150–250 mg m$^{-2}$ and orally 150–300 mg twice daily on days 3 and 4. Cardiovascular effects of nifedipine were dose limiting and the maximum tolerated dose was 60 mg bid. Mean area under the plasma concentration curve ($AUC_{0\text{-}\infty}$) and plasma half-life ($\beta$) of nifedipine and its major metabolite MI at the highest dose level were 7.87 $\mu$M.h, 7.97 h and 4.97 $\mu$M.h, 14.0 h respectively. Nifedipine did not interfere with the pharmacokinetics of etoposide.

Resistance to chemotherapy precludes successful therapy of most solid tumours. One mechanism of resistance to multiple cytotoxics is multidrug resistance or MDR and is mediated by an energy dependent efflux membrane pump. It is characterised by the expression of P-glycoprotein (P-gp) on the surface of malignant cells. Once resistance occurs it is exhibited against a range of cytotoxics including vinca alkaloids, epidopophyllotoxins, anthracyclines, and colchicine (Moscow & Cowan, 1988). *In vitro* studies have shown that the expression of the P-gp is strongly correlated with reduced intracellular accumulation of cytotoxic drugs and resistance to their action (Endicott & Ling, 1989). It has now been shown that several human tumours express P-gp and this may especially be apparent in tumours derived from tissues that normally express high levels of P-gp (e.g. kidney) (Bell *et al.*, 1985; Fojo *et al.*, 1987; Gerlach *et al.*, 1987; Ma *et al.*, 1987). It has also been shown that some haematological malignancies which progress after an initial response to chemotherapy, show significantly increased expression of P-gp compared to pre-treatment levels and may exhibit an improved response with the use of verapamil as modifier of MDR (Dalton *et al.*, 1989).

Several drugs act as modulators of MDR by competitively binding to P-gp and inhibiting the efflux of cytotoxic drugs from the cells. To achieve reversal of MDR *in vivo* may however require doses of modulators near to or even exceeding the maximum tolerated doses of those drugs leading to unacceptable toxicity. However, short term use of modifiers may allow higher levels to be achieved temporarily. In addition to the dose of the modifier, the extent of plasma protein binding determines the available free fraction of drugs for competitive binding to the P-gp. In one previous study the extent of binding of a modulator to $\alpha_1$-acid glycoprotein was inversely related to the reduction in the reversal of MDR *in vitro* (Chatterjee *et al.*, 1990). In addition to modification of MDR, modifiers of drug resistance may be effective through other potential mechanisms. It is also important to consider the role of metabolites in MDR modulation (Merry *et al.*, 1989) and potential pharmacokinetic and pharmacodynamic interactions between MDR modifiers and cytotoxic drugs (Kerr *et al.*, 1986).

Correspondence: P.A. Philip, ICRF Clinical Oncology Unit, Churchill Hospital, Oxford OX3 7LJ, UK.
Received 15 May 1991; and in revised form 15 October 1991.

Nifedipine is a dihydropyridine calcium channel blocker commonly used in the treatment of ischaemic heart disease and hypertension. It is metabolised in the liver by the microsomal mixed function oxidase, cytochrome P-450 IIIA, to the pyridine metabolite M I (Schellens *et al.*, 1988). Protein binding of nifedipine is 92–98%.

Etoposide is a broad spectrum cytotoxic drug whose biotransformation is partly dependent on the cytochrome P-450 dependent microsomal enzymes (Haim *et al.*, 1987). DNA damage and cytotoxicity correlate well with the intracellular concentrations of etoposide. Yalowich and Ross (1985) showed that verapamil increased the intracellular levels of etoposide in L1210 cells *in vitro*, and this elevation of levels was linearly correlated with enhancement of DNA damage and cytotoxicity. The increase in the intracellular levels was due to inhibition of efflux of etoposide from the cells. Chao *et al.* (1990) showed that cyclosporin increases the cytotoxicity of etoposide to MDR expressing leukaemic cells by nearly 20-fold.

Nifedipine was chosen for this study because: (1) relative lack of cardiac toxicity compared to verapamil which has been commonly employed in modulating MDR in clinical studies, (2) nifedipine may reverse MDR at concentrations which may be achiveable *in vivo* (Mickisch *et al.*, 1990), (3) nifedipine may reverse resistance to cytotoxics (e.g. cisplatin) by other mechanisms as well as reversal of MDR (Onoda *et al.*, 1986).

The aims of this phase I study were to determine (1) the maximum tolerated dose of nifedipine in patients receiving single agent etoposide, (2) pharmacokinetics of nifedipine at the different dose levels, (3) pharmacodynamic and pharmacokinetic interactions between nifedipine and etoposide and (4) tumour response.

### Materials and methods

### *Patients*

Approval to conduct this study was granted by The Central Oxford Research Ethical Committee. Fifteen patients (nine males and six females) with a mean age of 46.2 years (28–69 years), participated in this study after giving informed consent. Patients had a performance status of ECOG 0–2 and no history of cardiovascular disease. An electrocardiogram was performed on all patients prior to entry into the study in order to exclude patients with recent myocardial ischaemia or conduction defects.

Patients had histologically confirmed malignancy: renal cell carcinoma five, breast carcinoma three, hepatocellular carcinoma 3, ovarian adenocarcinoma two, melanoma one, soft tissue sarcoma one. Eight patients had received prior chemotherapy, and four had received hormonal treatment.

### *Treatment*

Nifedipine slow release (Bayer, UK) was administered orally for 6 days at three dose levels: 40 mg bid, 60 mg bid and 80 mg bid. If one dose level was tolerated well then the dose was escalated with only one escalation allowed per patient. To assess any interactions between nifedipine and etoposide, nifedipine on days 1 and 2 was omitted from either the first or second course of therapy.

Etoposide (Vepesid, Bristol-Myers-Squibb, USA) was administered at a starting dose of 150 mg $m^{-2}$ in 1/2 l normal saline 0.9% intravenoulsy (i.v.) over 45 min on day 2, 2 h from the previous dose of nifedipine, followed by 150 mg bid po on days 3 and 4.

The plan was to increase the dose of etoposide after six patients have been treated without evidence of significant toxicity. The dose of etoposide was escalated to 200 mg $m^{-2}$ i.v. on day 2 and 200–300 mg bid po on days 3 and 4.

Treatment was administered on outpatient basis except for the intravenous etoposide which necessitated an overnight admission to hospital.

### *Etoposide pharmacokinetics*

Etoposide pharmacokinetics were determined following intravenous administration of two courses of treatment given with and without nifedipine. Ten ml of venous blood was withdrawn at mid-infusion and then 0 min, 15 min, 30 min, 45 min, 60 min, 2 h, 4 h, 6 h, 9 h, 12 h and 24 h from the end of the infusion. Plasma was immediately separated and stored at −20°C pending analysis. Etoposide levels were determined by reverse-phase high-performance liquid chromatography with UV detection (Harvey *et al.*, 1985). Pharmacokinetic parameters were derived using STRIPE, an interactive computer programme (Johnson & Woollard, 1983). The area under the plasma concentration curve was extrapolated to infinity.

### *Nifedipine pharmacokinetics*

Nifedipine pharmacokinetics were determined following the third dose of nifedipine of the first course of treatment to allow for the attainment of a near-steady concentration in plasma. Venous blood was obtained 2, 3, 4, 6, and 8 h post-dosing. Ten ml of blood were collected into a heparinised tube protected from light with aluminum foil. Plasma was separated under sodium lighting and stored at −20°C pending analysis. Nifedipine and the MI metabolite were determined by capillary gas chromatography using a modification of the method of Schmid *et al.* (1988). Pharmacokinetic analysis was performed using an in-house computer programme (N. Oates, St Mary's Hospital, London) fitting the data into a non-linear two-compartment model. Area under plasma concentration (AUC) of nifedipine and its MI metabolite were extrapolated to infinity ($AUC_{0-\infty}$) using the respective half-life values.

### *Statistical analysis*

The pharmacokinetic parameters of etoposide in the group of ten patients studied with and without nifedipine were compared using the Wilcoxon signed ranks matched pairs test. Statistical significance was taken at a $P$ value of $\leqslant 0.05$.

### Results

### *Nifedipine pharmacokinetics*

Table I shows the peak plasma concentration, $AUC_{0-\infty}$ and plasma half-life ($\beta$) of nifedipine and its MI metabolite at the three dose levels. Peak plasma concentrations of nifedipine were 0.45 μM, 0.60 μM and 0.66 μM for the three dose levels. Peak MI metabolite concentration in plasma were 0.41 μM, 0.38 μM and 0.47 μM at the corresponding dose levels.

### *Etoposide pharmacokinetics and interactions with nifedipine*

Pharmacokinetic parameters of etoposide following intravenous administration were studied in ten patients for any possible interactions with nifedipine (Table II). The terminal half-life ($\beta$), $AUC_{0-\infty}$, $AUC_{0-\infty}$/100 mg dose, total plasma clearance (Cl), and volume of distribution (Vd) of etoposide were similar to those observed in a larger population study (Joel S., personal communication). Pre-treatment with nifedipine did not significantly alter any of those parameters implying lack of pharmacokinetic interactions between the two drugs ($P > 0.05$).

### *Nifedipine toxicity*

The side-effects of nifedipine which were represented the dose-limiting end-points were severe headaches, postural hypotension and postural dizziness. Severe headache was that requiring regular analgesia and markedly interfering with the normal daily routine. Dose-limiting postural hypotension was defined as >20 mmHg drop in the systolic blood pressure when assuming the upright posture with or without postural dizziness. Postural dizziness was defined as the sensation of light-headedness particularly associated with the upright posture which interfered with normal activity.

**Table II** Comparison of etoposide (Et) pharmacokinetic parameters in ten patients investigated with and without nifedipine (NF)

| *Parameter* | *Et alone* | *Et plus NF* | *P (Wilcoxon)* |
|---|---|---|---|
| $\beta$ T1/2 (h) | 4.67 (±1.3) | 4.7 (±1.5) | 0.61 |
| $AUC_{0-\infty}$/100 mg (mg/ml.h) | 55.8 (±17.2) | 50.78 (±16.4) | 0.31 |
| Cl (ml/min) | 35.2 (±18.6) | 37.3 (±17.7) | 0.42 |
| Vd (L) | 13.1 (±4.2) | 14.1 (±4.5) | 0.58 |

Mean values are given with standard deviations in parentheses.

**Table I** Pharmacokinetic parameters of nifedipine and its MI metabolite in ten patients after the oral administration of nifedipine slow release preparation

| *Dose* | *n* | *Nifedipine* $C_{max}$ *(μM)* | *Nifedipine* $AUC_{0-\infty}$ *(μM.h)* | *Nifedipine T1/2(β) (h)* | *MI metabolite* $C_{max}$ *(μM)* | *MI metabolite* $AUC_{0-\infty}$ *(μM.h)* | *MI metabolite T1/2(β) (h)* |
|---|---|---|---|---|---|---|---|
| 40 mg bid | 2 | 0.45 (0.39–0.50) | 2.86 (2.45–3.26) | 4.35 (3.7–5.0) | 0.41 (0.22–0.59) | 3.06 (2.74–3.38) | 19.7 (2.3–37.0) |
| 60 mg bid | 5 | 0.60 (0.40–0.70) | 7.02 (2.58–19.0) | 8.77 (3.6–19.3) | 0.38 (0.18–0.61) | 2.39 (0.92–4.93) | 8.5 (2.8–23.9) |
| 80 mg bid | 3 | 0.66 (0.42–0.80) | 7.87 (4.01–14.1) | 7.97 (3.0–12.2) | 0.47 (0.36–0.55) | 4.97 (2.21–7.1) | 14.0 (2.0–25.8) |

Mean values are shown with the range in parentheses.

Seven, five and two patients were entered into the 40 mg, 60 mg and 80 mg dose levels respectively. The dose of nifedipine was escalated in four patients who were entered at the 40 mg and 60 mg dose levels respectively. The number of courses of nifedipine at each of the three doses were 14, 13 and 8 respectively. Following dose escalations, eight, eight and six patients were treated at each of the those dose levels respectively. None of the eight subjects who received nifedipine at the 40 mg dose level experienced side effects, while one out of eight patients on the 60 mg dose level experienced symptomatic postural hypotension necessitating either a dose reduction of nifedipine. Three of the six patients on the 80 mg dose level experienced significant toxicity which necessitated dose reductions in one and discontinuation of nifedipine in two (Table III). The degree of toxicity at the 80 mg dose level indicated that the maximum tolerated dose (MTD) of nifedipine in an outpatient setting is 60 mg bid.

*Combination toxicity*

Two patients were withdrawn from analysis due to early death due to disease progression after one course of chemotherapy and before proper evaluation for toxicity. There were no treatment related deaths and overall toxicity was moderate and expected with the dose and schedule of etoposide employed. Only two patients had severe myelosuppression complicated by sepsis. Side effects related to modulation of MDR in normal tissues (e.g. diarrhoea) were absent. Table IV gives details of combination toxicity in the nine patients who received the higher dose of etoposide.

*Response*

Of the 15 patients studied 12 exhibited progressive disease, two had stable disease with a mean duration of 2 months, one patient achieved a mixed response, with partial remission in lung metastases and disease progression in the orbital cavity.

**Discussion**

Peak plasma concentrations of nifedipine achieved in this study were less than 1 μM. *In vitro* work on the modulation of MDR by nifedipine has employed concentrations above 5 μM in cell lines exhibiting levels of resistance which are probably much higher than those encountered in clinical conditions (Kessel & Wilberding, 1984; Willingham *et al.*, 1986). However, it has recently been demonstrated by Mickisch *et al.* (1990) that concentrations of nifedipine as low as 1 μM could significantly enhance cytotoxicity by vinblastine *in vitro* to cells from fresh human renal cell carcinoma. Concentrations up to 0.47 μM of the MI metabolite were achieved in plasma. There is no information on the activity of this metabolite in reversing MDR *in vitro*. If it was possible to show modulation of MDR by the MI metabolite then this would increase the effective plasma concentrations to modulate MDR by 50%. Further studies are required to ascertain the contribution of the MI metabolite to the reversal of MDR *in vitro* since relatively significant plasma concentrations are achieved. The half-life of the MI metabolite was shorter in some of the patients and that may be explained on the basis of metabolism by enzymes different to those metabolising nifedipine.

Verapamil is another calcium channel blocker which has been clinically investigated for its MDR modulating properties. Unfortunately its clinical use has been associated with signifciant cardiovascular toxicity in the form of hypotension and/or prolonged atrioventricular conduction time. There was also interaction with the clearance of doxorubicin (Kerr *et al.*, 1986). The present study shows that cardiovascular (i.e. calcium blocking) effects of nifedipine are the dose-

**Table III** Nifedipine toxicity profile in patients at the three dose levels who experienced severe toxicity

| *Dose (bid)* | *n* | *Hypoten* | *Dizziness* | *Headache* | *Dose reduct* |
|---|---|---|---|---|---|
| 40 mg | 8 | 0 | 0 | 0 | 0 |
| 60 mg | 8 | 1 | 2 | 1 | 1 |
| 80 mg | 6 | 2 | 3 | 3 | 2 |

**Table IV** Toxicity data on the combination therapy in nine patients on the higher dose level of etoposide

| | *WHO Grade* | | | | |
|---|---|---|---|---|---|
| *Toxicity* | *0* | *1* | *2* | *3* | *4* |
| Anaemia | 6 | 2 | 1 | 0 | 0 |
| Leucopenia | 2 | 2 | 2 | 2 | 1 |
| Thrombocytopenia | 8 | 0 | 0 | 1 | 0 |
| Infection | 7 | 0 | 1 | 1 | 0 |
| Alopecia | 2 | 1 | 5 | 1 | 0 |
| Nausea/Vomiting | 8 | 0 | 1 | 0 | 0 |
| Diarrhoea | 9 | 0 | 0 | 0 | 0 |

limiting factor in therapy. Toxicity of nifedipine is clearly dose related (Table IV) and the maximum tolerated dose of nifedipine for future studies should be 60 mg bid in an outpatient setting.

Etoposide was commenced on day 2 of the cycle because it was intended to treat the patient with nifedipine for the least possible time to minimise toxicity. With the calculated nifedipine plasma half-life of 6–8 h, approximately 90% of the steady plasma concentrations are expected to occur within 26 h of starting nifedipine. The starting dose of etoposide was chosen on the bais of patient safety in order to avoid unexpected synergy with nifedipine. After the initial cohort of six patients it was considered safe to escalate the dose of etoposide because it is prudent to investigate modulation of etoposide toxicity by nifedipine at therapeutic doses of etoposide.

There was no change in the pharmacokinetic parameters of etoposide when used in combination with nifedipine. There is also no indication in this study that nifedipine has potentiated the toxicity of etoposide. In particular there were no features suggesting increased toxicity of etoposide to tissues rich in P-gp such as the gastrointestinal tract. Detailed studies to investigate cardiac toxicity were not performed as the dose limiting toxicity of nifedipine was due to symptoms predicted from its mode of action. We did not undertake detailed cardiac investigations to determine interactions between etoposide and nifedipine because cardiotoxicity due to etoposide is very unusual. Nifedipine on the other hand has no obvious effect on atrioventricular conduction compared to verapamil (Rowland *et al.*, 1979).

There were no tumour responses in this present phase I study except for one patient with breast carcinoma who had a mixed response. Response to etoposide would be expected to depend on (a) response of the tumour type to the chemotherapy used, (b) degree of importance of MDR in drug resistance in the tumours studied, (c) achievement of plasma nifedipine levels sufficient to reverse MDR, and (d) the contribution of other mechanisms of resistance to etoposide such as reduced topoisomerase II activity tumour cells (Beck, 1989).

Hu *et al.* (1990) have shown that cyclosporin and verapamil exhibited synergism in reversing MDR *in vitro* at concentrations normally seen in clinical situations. In future studies a combination of MDR modulators may potentially be employed, each with different dose limiting toxicity to test whether there would be synergy in their modification of MDR.

### References

BECK, W.T. (1989). Unknotting the complexities of multidrug resistance: the involvement of DNA topoisomerase in drug action and resistance. *JNCI*, **81**, 1683.

BELL, D.R., GERLACH, J.H., KARTNER, N., BUICK, R.N. & LING, V. (1985). Detection of P-glycoprotein in ovarian cancer: a molecular marker associated with multidrug resistance. *J. Clin. Oncol.*, **3**, 311.

CHAO, N.J., AIHARA, M., BLUME, K.G. & SIKIC, B.I. (1990). Modulation of etoposide (VP-16) cytotoxicity by verapamil or cyclosporine in multidrug-resistant human leukemic cell lines and normal bone marrow. *Exp. Hematol.*, **18**, 1193.

CHATTERJEE, M., ROBSON, C.N. & HARRIS, A.L. (1990). Reversal of multidrug resistance by verapamil and modulation by $\alpha_1$-acid glycoprotein in wild-type and multidrug-resistant chinese hamster ovary cell lines. *Cancer Res.*, **50**, 2818.

DALTON, W.S., GROGAN, T.M., MELTZER, P.S., SCHEPER, R.J., DIVINE, B.G. & TAYLOR, C.W. (1989). Drug-resistance in multiple myeloma and non-Hodgkins's lymphoma: detection of p-glycoprotein and potential circumvention by addition of verapamil to chemotherapy. *J. Clin. Oncol.*, **7**, 415.

ENDICOTT, J.A. & LING, V. (1989). The biochemistry of P-glycoprotein-mediated multidrug resistance. *Annu. Rev. Biochem.*, **58**, 137.

FOJO, A.T., SHEN, D.-W., MICKLEY, L.A., PASTAN, I. & GOTTESMAN, M.M. (1987). Intrinsic drug resistance in human kidney cancer is associated with expression of a human multidrug-resistance gene. *J. Clin. Oncol.*, **5**, 1922.

GERLACH, J.H., BELL, D.R. & KARAKOUSIS, C. (1987). P-glycoprotein in human sarcoma: evidence for multidrug resistance. *J. Clin. Oncol.*, **5**, 1452.

HAIM, N., NEMEC, J., ROMAN, J. & SINHA, B.K. (1987). *In vitro* metabolism of etoposide (VP16-213) by liver microsomes and irreversible binding of reactive intermediates to microsomal proteins. *Biochem. Pharmacol.*, **36**, 527.

HARVEY, V.J., JOEL, S.P., JOHNSTON, A. & SLEVIN, M.L. (1985). High performance liquid chromatography of etoposide in plasma and urine. *J. Chromatogr.*, **339**, 419.

HU, X.F., MARTIN, T.J., BELL, D.R., DE LUISE, M. & ZALCBERG, J.R. (1990). Combined use of cyclosporin A and verapamil in modulating multidrug resistance in human leukemia cell lines. *Cancer Res.*, **50**, 2953.

JOHNSON, A. & WOOLLARD, R.C. (1983). STRIPE: an interactive computer programme for the analysis of drug pharmacokinetics. *J. Pharmacol. Methods*, **9**, 193.

KERR, D.J., GRAHAM, J., CUMMINGS, J. & 4 others (1986). The effect of verapamil on the pharmacokinetics of adriamycin. *Cancer Chemother. Pharmacol.*, **18**, 239.

KESSEL, D. & WILBERDING, C. (1984). Interactions between calcium antagonists, calcium fluxes and anthracycline transport. *Cancer Lett.*, **25**, 97.

MA, D.D., SCURR, R.D., DAVEY, R.A. & 5 others (1987). Detection of a multidrug resistant phenotype in acute non-lymphoblastic leukaemia. *Lancet*, **i**, 135.

MERRY, S., FLANIGAN, P., SCHLICK, E., FRESHNEY, R.I. & KAYE, S.B. (1989). Inherent adriamycin resistance in a murine tumour line: circumvention with verapamil and norverapamil. *Br. J. Cancer*, **59**, 895.

MICKISCH, G.H., KOSSIG, J., KEILHAUER, G., SCHLICK, E., TSCHADA, R.K. & ALKEN, P.M. (1990). Effects of calcium antagonists in multidrug resistant primary human renal cell carcinomas. *Cancer Res.*, **50**, 3670.

MOSCOW, J.A. & COWAN, K.H. (1988). Multidrug resistance. *JNCI*, **80**, 14.

ONODA, J.M., JACOBS, J.R., TAYLOR, J.D., SLOANE, B.F. & HONN, K.V. (1986). Cisplatin and nifedipine: synergistic cytoxicity against murine solid tumours and their metastases. *Cancer Lett.*, **30**, 181.

ROWLAND, E., EVANS, T. & KRIKLER, D. (1979). Effect of nifedipine on atrioventricular conduction as compared with verapamil. *Br. Heart J.*, **42**, 124.

SCHELLENS, J.H.M., SOONS, P.A. & BREIMER, D.D. (1988). Lack of bimodality in nifedipine plasma kinetics in a large population of healthy subjects. *Biochem. Pharmacol.*, **37**, 2507.

SCHMID, B.J., PERRY, H.E. & IDLE, J.R. (1988). Determination of nifedipine and its three principal metabolites in plasma and urine by automated electron-capture capillary gas chromatography. *J. Chromatogr. Biomed. Applic.*, **425**, 107.

WILLINGHAM, M.C., CORNWELL, M.M., CARDARELLI, C.O., GOTTESMAN, M.M. & PASTAN, I. (1986). Single cell analysis of daunomycin uptake and efflux in multidrug-resistant and sensitive KB cells: effects of verapamil and other drugs. *Cancer Res.*, **46**, 5941.

YALOWICH, J.C. & ROSS, W.E. (1985). Verapamil-induced augmentation of etoposide accumulation in L1210 cells *in vitro*. *Cancer Res.*, **45**, 1651.


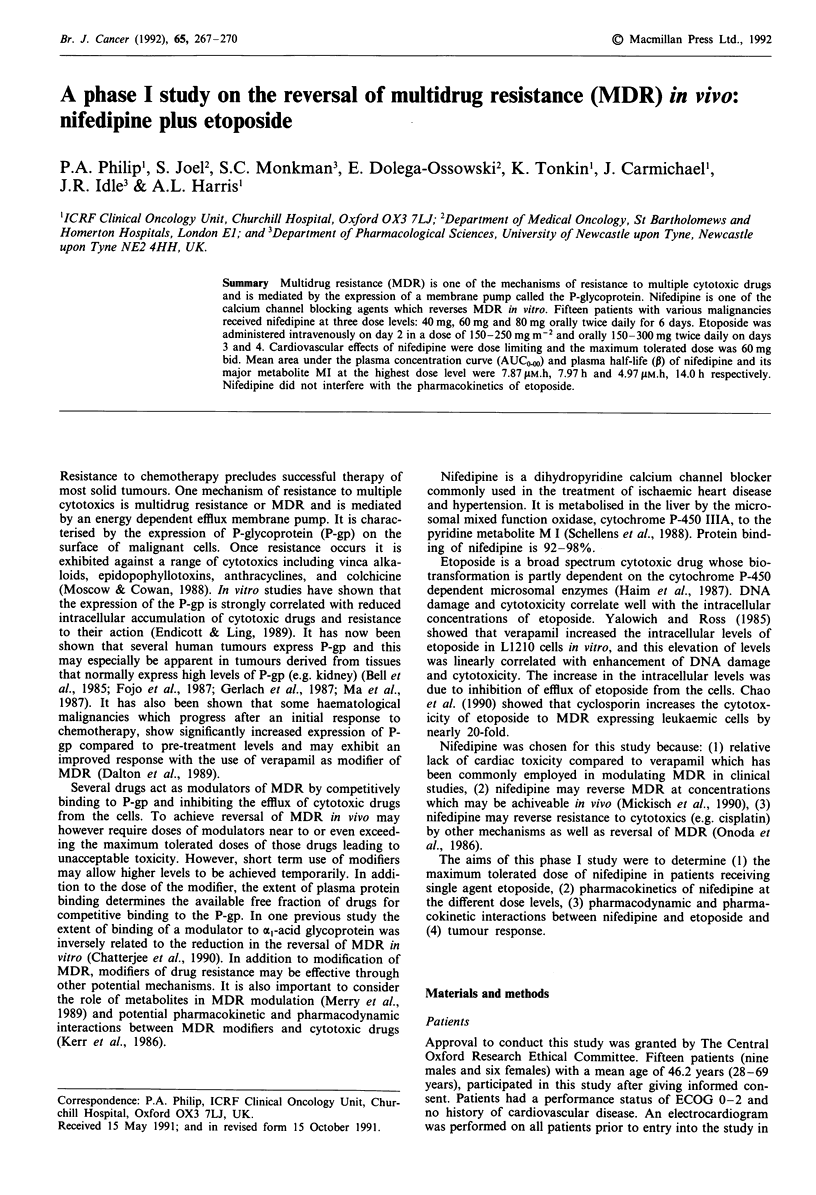

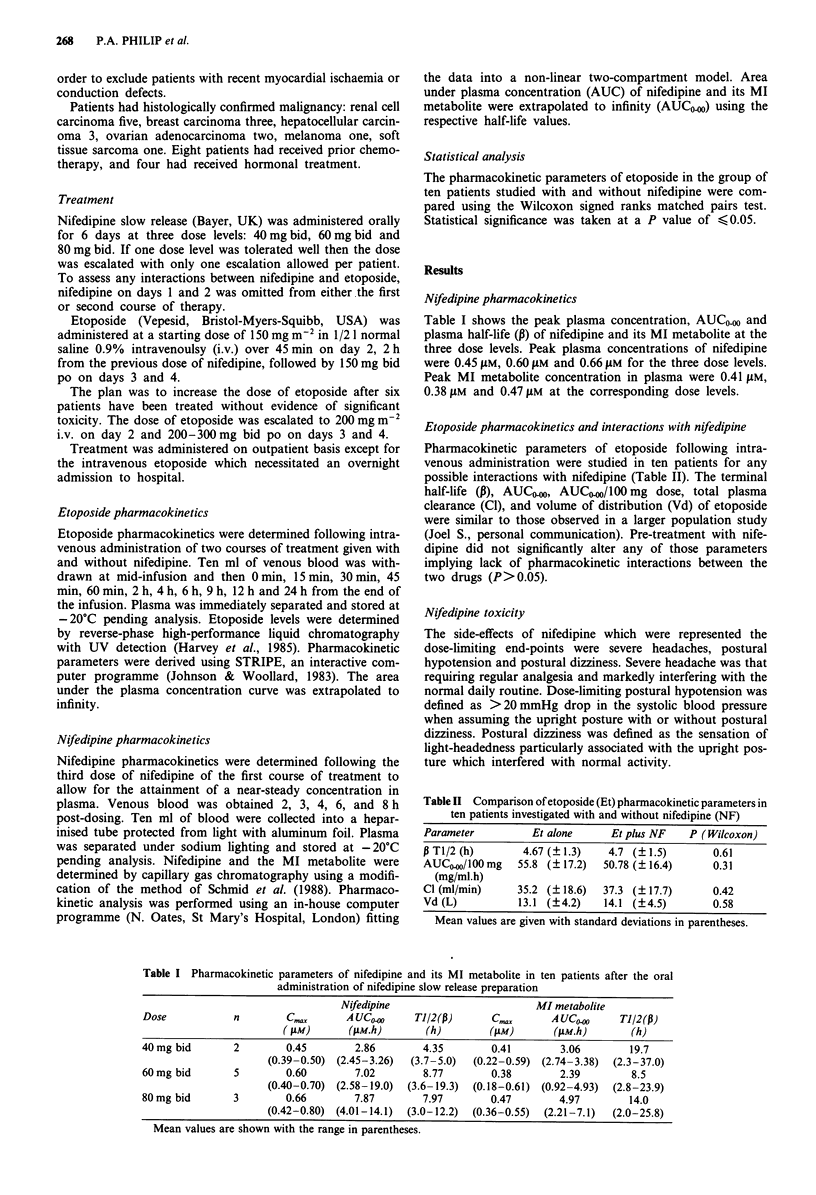

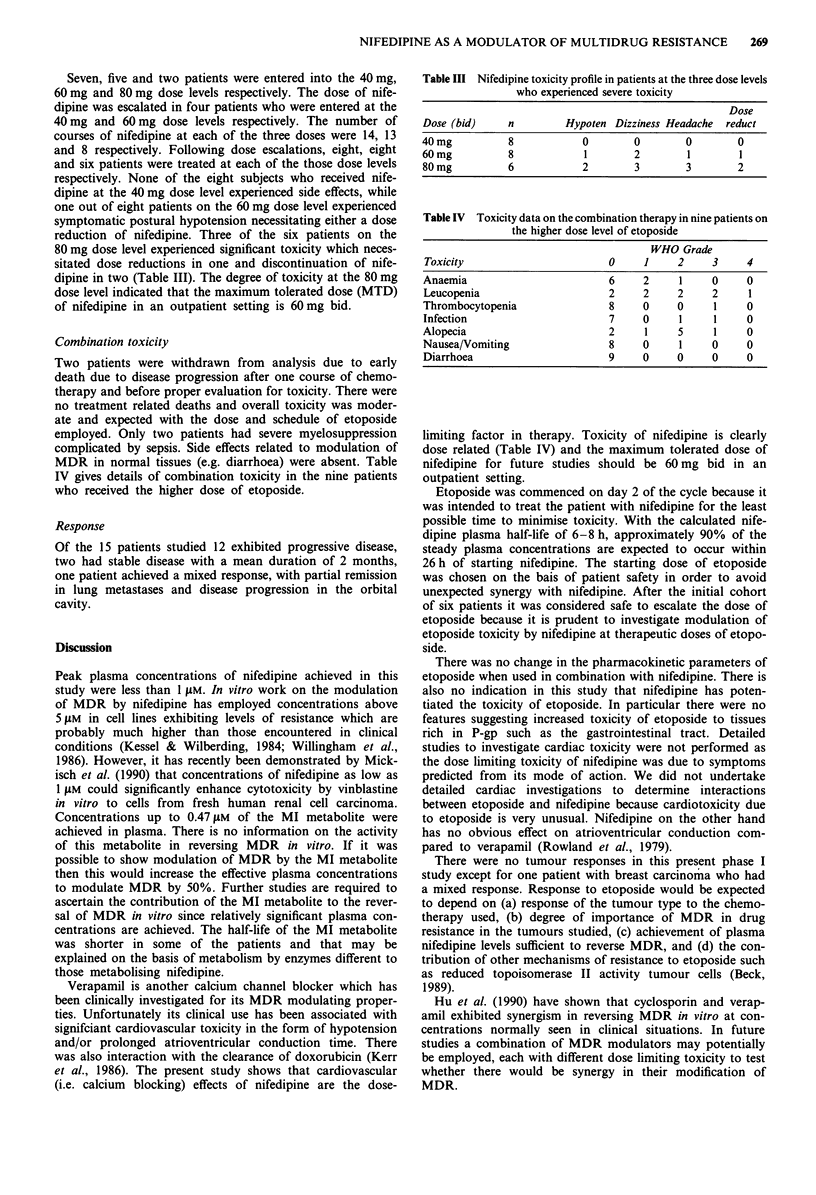

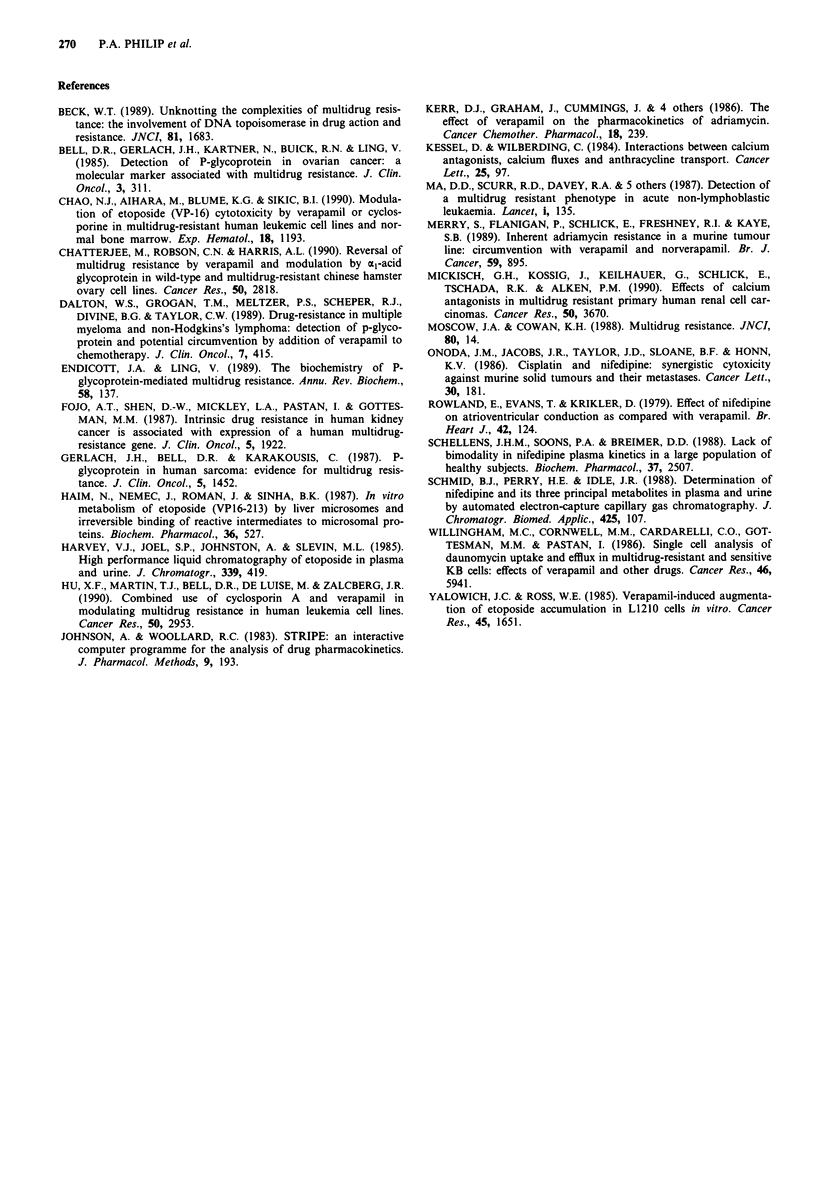


## References

[OCR_00435] Beck W. T. (1989). Unknotting the complexities of multidrug resistance: the involvement of DNA topoisomerases in drug action and resistance.. J Natl Cancer Inst.

[OCR_00440] Bell D. R., Gerlach J. H., Kartner N., Buick R. N., Ling V. (1985). Detection of P-glycoprotein in ovarian cancer: a molecular marker associated with multidrug resistance.. J Clin Oncol.

[OCR_00446] Chao N. J., Aihara M., Blume K. G., Sikic B. I. (1990). Modulation of etoposide (VP-16) cytotoxicity by verapamil or cyclosporine in multidrug-resistant human leukemic cell lines and normal bone marrow.. Exp Hematol.

[OCR_00452] Chatterjee M., Robson C. N., Harris A. L. (1990). Reversal of multidrug resistance by verapamil and modulation by alpha 1-acid glycoprotein in wild-type and multidrug-resistant Chinese hamster ovary cell lines.. Cancer Res.

[OCR_00458] Dalton W. S., Grogan T. M., Meltzer P. S., Scheper R. J., Durie B. G., Taylor C. W., Miller T. P., Salmon S. E. (1989). Drug-resistance in multiple myeloma and non-Hodgkin's lymphoma: detection of P-glycoprotein and potential circumvention by addition of verapamil to chemotherapy.. J Clin Oncol.

[OCR_00465] Endicott J. A., Ling V. (1989). The biochemistry of P-glycoprotein-mediated multidrug resistance.. Annu Rev Biochem.

[OCR_00472] Fojo A. T., Shen D. W., Mickley L. A., Pastan I., Gottesman M. M. (1987). Intrinsic drug resistance in human kidney cancer is associated with expression of a human multidrug-resistance gene.. J Clin Oncol.

[OCR_00476] Gerlach J. H., Bell D. R., Karakousis C., Slocum H. K., Kartner N., Rustum Y. M., Ling V., Baker R. M. (1987). P-glycoprotein in human sarcoma: evidence for multidrug resistance.. J Clin Oncol.

[OCR_00481] Haim N., Nemec J., Roman J., Sinha B. K. (1987). In vitro metabolism of etoposide (VP-16-213) by liver microsomes and irreversible binding of reactive intermediates to microsomal proteins.. Biochem Pharmacol.

[OCR_00487] Harvey V. J., Joel S. P., Johnston A., Slevin M. L. (1985). High-performance liquid chromatography of etoposide in plasma and urine.. J Chromatogr.

[OCR_00492] Hu X. F., Martin T. J., Bell D. R., de Luise M., Zalcberg J. R. (1990). Combined use of cyclosporin A and verapamil in modulating multidrug resistance in human leukemia cell lines.. Cancer Res.

[OCR_00498] Johnston A., Woollard R. C. (1983). STRIPE: an interactive computer program for the analysis of drug pharmacokinetics.. J Pharmacol Methods.

[OCR_00503] Kerr D. J., Graham J., Cummings J., Morrison J. G., Thompson G. G., Brodie M. J., Kaye S. B. (1986). The effect of verapamil on the pharmacokinetics of adriamycin.. Cancer Chemother Pharmacol.

[OCR_00508] Kessel D., Wilberding C. (1984). Interactions between calcium antagonists, calcium fluxes and anthracycline transport.. Cancer Lett.

[OCR_00513] Ma D. D., Scurr R. D., Davey R. A., Mackertich S. M., Harman D. H., Dowden G., Isbister J. P., Bell D. R. (1987). Detection of a multidrug resistant phenotype in acute non-lymphoblastic leukaemia.. Lancet.

[OCR_00518] Merry S., Flanigan P., Schlick E., Freshney R. I., Kaye S. B. (1989). Inherent adriamycin resistance in a murine tumour line: circumvention with verapamil and norverapamil.. Br J Cancer.

[OCR_00524] Mickisch G. H., Kössig J., Keilhauer G., Schlick E., Tschada R. K., Alken P. M. (1990). Effects of calcium antagonists in multidrug resistant primary human renal cell carcinomas.. Cancer Res.

[OCR_00530] Moscow J. A., Cowan K. H. (1988). Multidrug resistance.. J Natl Cancer Inst.

[OCR_00534] Onoda J. M., Jacobs J. R., Taylor J. D., Sloane B. F., Honn K. V. (1986). Cisplatin and nifedipine: synergistic cytotoxicity against murine solid tumors and their metastases.. Cancer Lett.

[OCR_00540] Rowland E., Evans T., Krikler D. (1979). Effect of nifedipine on atrioventricular conduction as compared with verapamil. Intracardiac electrophysiological study.. Br Heart J.

[OCR_00545] Schellens J. H., Soons P. A., Breimer D. D. (1988). Lack of bimodality in nifedipine plasma kinetics in a large population of healthy subjects.. Biochem Pharmacol.

[OCR_00550] Schmid B. J., Perry H. E., Idle J. R. (1988). Determination of nifedipine and its three principal metabolites in plasma and urine by automated electron-capture capillary gas chromatography.. J Chromatogr.

[OCR_00558] Willingham M. C., Cornwell M. M., Cardarelli C. O., Gottesman M. M., Pastan I. (1986). Single cell analysis of daunomycin uptake and efflux in multidrug-resistant and -sensitive KB cells: effects of verapamil and other drugs.. Cancer Res.

[OCR_00563] Yalowich J. C., Ross W. E. (1985). Verapamil-induced augmentation of etoposide accumulation in L1210 cells in vitro.. Cancer Res.

